# Book Notices

**Published:** 1889-11

**Authors:** 


COMPARATIVE ANATOMY AND PHYSIOLOGY.—By Jeffrey Bell, M. A., Professor of Comparative Anatomy at King's College, illustrated with 229 engravings. Philadelphia. Lea Brothers & Co., 1885.

This is a condensed yet comprehensive handbook on Comparative Anatomy, in which the subject is brought well up to recent observations, constituting a good manual for zoological study.

Opening up with a description of the lowest forms of life as found in protoplasm it merges to an account of the general structure of animals, including the organs of digestion; the blood and vascular system; of respiration; of the organs of excretion and secretion; organs of movement; vocal organs; organs of reproduction, etc. The illustrations are numerous and beautiful, and of exceeding interest to the physiologist and the student of science.

A TEXT-BOOK OF ANIMAL PHYSIOLOGY, with introductory chapters on General Biology, and a full treatment of Reproduction for students of Human and comparative medicine and of General Biology, by Wesley Mills, M. A., M. D., L. R. C. P. (Eng.), Professor of Physiology in McGill University and the veterinary College, Montreal; with over 500 illustrations. New York. D. Appleton & Co., 1889.

Prof. Mills has here presented us a work of exceeding interest, and though compact is sufficiently full, being a work of 690 large octavo pages, neat and excellent in its typographical execution, and profusely illustrated.

To the student of General Biology it must prove exceedingly interesting and instructive.

The summary with which each chapter concludes will be acceptable to both teacher and student; and the "Special Considerations" toward the close are replete with matter of instruction and interest. This book will take a high place among the standard works of the day.

WARNER'S THERAPEUTIC REFERENCE BOOK. Philadelphia. Wm. R. Warner & Co., 1889. A snug little book of over 120 pages, containing much information on various subjects of special usefulness and interest to the physician. Amongst which we mention weights and measures; Prescription writing; posological table; Aid in memorizing doses; Hypodermic doses and formulæ; Incompatibles; Poisons and their antidotes; Asphyxia and its treatment; Signs of pregnancy; Post mortems; Syrups, Elixirs, Coated pills, Parvules, and numerous useful and valuable preparations and formulæ. Price, $1.00.

THE PSYCHOLOGY OF ATTENTION. By Th. Ribot. Translated by J. Fitzgerald, New York: The Humboldt Publishing Co., 28 Lafayette Place. Paper, Price 15 cents. Another interesting monograph by the same distinguished author who has already enriched the literature of psychology with three very remarkable works on "The Diseases of the will," "The Diseases of Memory," and "The Diseases of Personalty" respectively. The works named have been translated into English, and are published in "The Humboldt Library," Like them, the present work is a study of very recondite problems of psychology—the nature and workings of the mind of man—presented in language understandable by every intelligent reader. That it is instructive "goes without saying." But furthermore, it is highly entertaining. In the series of works to which it belongs are found illustrations of abnormal psychic states more striking than the "double personality" portrayed in "Dr. Jekyll and Mr. Hyde."

THE PHYSIOLOGY OF THE DOMESTIC ANIMALS.—A Text-Book for veterinary and medical Students and practitioners, by Rob't. Meade Smith, A. M., M. D., Professor of Comparative Physiology in the University of Pennsylvania; Fellow of the College of Physicians and of Academy of the Natural Sciences Philadelphia; of the American Physiological Society; of the American Society of Naturalists; Associe etranger de la Societe Francaise de Hygiene, etc. With over four hundred illustrations. Philadelphia and London. F. A. Davis, publisher, 1889.

This is a very ably written and beautifully illustrated work of 938 large oc. pages. It is the only work of the kind in the English language, and will be gladly welcomed by progressive minds in this country. There is certainly a need for such a work, and it is a matter of surprise that we have not had something on the subject before. It furnishes certainly a wide and comparatively unexplored field for study and observation. Much that we know of the effects of drugs and of life in the higher animals has been acquired through vivisection and experiments on the lower animals, and the more we learn of the vital operations in lower mammals, the more we shall be able to comprehend the laws and functions that prevail in the higher forms.

Under the head of General Physiology the author treats in a very clear and able manner of the Physiology of Animal Cells; of Cellular physics, Cellular Chemistry; the Chemical Processes in Cells, etc. In part second he treats of the Nutritive Functions, Foods, Digestion, Deglutition, Rumination, Absorption. The Blood; Respiration; the mammary Secretion, Cutaneous Functions, Nutrition; Food required by the Herbivora under different conditions; Thirst, Animal heat; the Physiology of movement, etc.

The work is very full and satisfactory on the nervous system and the reproductive functions.

THE HOMILETIC REVIEW for October well sustains the high reputation of this minister's monthly. The leading paper is by Dr. Wayland Hoyt, being the closing half of his masterly presentation of his ideal Parish Minister. Seldom have we read a more suggestive article. Containing numerous interesting and instructive articles. The Exegetical papers are by Prof. Willis J. Beecher, and Drs. Chambers, Crosby and Gilmore. Dr. Stuckenberg's tribute to Prof. Christlieb will be read with tender interest. All the other departments are, as usual, full of valuable matter.

Published by FUNK & WAGNALLS, 18 and 20 Astor Place, New York. $3.00 per year; 30 cents per single number.

ESSENTIALS OF PHYSIOLOGY, arranged in the form of questions and answers prepared especially for students of medicine, by H. A. Hare, B. S. C., M. D. (University of Pa.) Demonstrator of Therapeutics and instructor in Physical Diagnosis in the Medical department, and instructor in Physiology in the Biological department of the University of Pennsylvania; Physician to St Agnes Hospital, etc.

Second edition. Thoroughly revised and enlarged. Philadelphia. W. B. Saunders, 913 walnut St. 1889. A very instructive and useful work for medical students·

